# Elevated VEGF Levels in Pulmonary Edema Fluid and PBMCs from Patients with Acute Hantavirus Pulmonary Syndrome

**DOI:** 10.1155/2012/674360

**Published:** 2012-08-22

**Authors:** Irina Gavrilovskaya, Elena Gorbunova, Frederick Koster, Erich Mackow

**Affiliations:** ^1^Department of Molecular Genetics and Microbiology, Stony Brook University, Stony Brook, NY 11794-5222, USA; ^2^Lovelace Respiratory Research Institute, 2425 Ridgecrest Dr. Albuquerque, NM 87108, USA

## Abstract

Hantavirus pulmonary syndrome is characterized by vascular permeability, hypoxia, and acute pulmonary edema. Vascular endothelial growth factor (VEGF) is induced by hypoxia, potently induces vascular permeability, and is associated with high-altitude-induced pulmonary edema. Hantaviruses alter the normal regulation of **β**3 integrins that restrict VEGF-directed permeability and hantavirus infected endothelial cells are hyperresponsive to the permeabilizing effects of VEGF. However, the role of VEGF in acute pulmonary edema observed in HPS patients remains unclear. Here we retrospectively evaluate VEGF levels in pulmonary edema fluid (PEF), plasma, sera, and PBMCs from 31 HPS patients. VEGF was elevated in HPS patients PEF compared to controls with the highest levels observed in PEF samples from a fatal HPS case. VEGF levels were highest in PBMC samples during the first five days of hospitalization and diminished during recovery. Significantly increased PEF and PBMC VEGF levels are consistent with acute pulmonary edema observed in HPS patients and HPS disease severity. We observed substantially lower VEGF levels in a severe HPS disease survivor after extracorporeal membrane oxygenation. These findings suggest the importance of patients' VEGF levels during HPS, support the involvement of VEGF responses in HPS pathogenesis, and suggest targeting VEGF responses as a potential therapeutic approach.

## 1. Introduction

Hantavirus Pulmonary Syndrome (HPS) is a hallmark capillary leak syndrome with a ~40% mortality rate, and Sin Nombre (SNV) is a prototypical HPS causing hantavirus associated with outbreaks of HPS disease in the Southwestern United States [1–5]. HPS is characterized by an acute febrile prodrome with thrombocytopenia rapidly progressing to acute pulmonary edema, hypoxia respiratory insufficiency, hypotension, and cardiogenic shock [1, 2, 5–7]. Hantaviruses predominantly infect the endothelial cell lining of vessels that form the primary fluid barrier of the vasculature. The pathogenesis of HPS is likely to result from the direct infection of pulmonary endothelial cells as well as hantavirus-induced responses of endothelial and immune cells. Immune cells are hypothesized to contribute to hantavirus disease through elevated levels of CD8^+^ T cells and cytokines such as TNF, yet the vascular endothelium is not disrupted in patients [5, 8–15]. *In vitro* SNV-infected endothelial cells are not permeabilized by infection alone or following the addition of TNF [14, 16], however pathogenic hantaviruses bind and inactivate *β*3 integrins which normally restrict VEGF-directed capillary permeability [17–21]. This nonlytic induction of vascular permeability suggests that hantaviruses alter normal responses of the endothelium to factors that elicit an edematous disease process [5–7, 10, 14, 16–18, 22–26]. 

A suggested role for VEGF in HPS was indicated by the VEGF enhanced permeability of capillaries within *β*3 integrin knockout mice [21, 27, 28] and findings that pathogenic hantaviruses similarly inhibit *β*_3_ integrin functions [17–19, 29]. Subsequent analysis demonstrated that hantavirus-infected endothelial cells are hyperresponsive to the permeabilizing effects of VEGF [18, 24, 30, 31]. VEGF is a cytokine that was first identified as a vascular permeability factor for its ability to potently induce edema ~50,000 times more effectively than histamine [32]. VEGF is induced by hypoxic conditions and causes high altitude pulmonary edema (HAPE) by binding to unique endothelial cell receptors that disrupt adherens junctions [33–39]. VEGF also transcriptionally induces hypoxia inducible factor-1*α* (HIF-1*α*) forming an autocrine loop that exacerbates permeability responses during hypoxia [36, 38–40]. The VEGF sensitivity of SNV infected endothelial cells, the role of VEGF in HAPE, and findings that HPS patients are acutely hypoxic prompted us to retrospectively evaluate VEGF levels in HPS patient samples. 

## 2. Materials and Methods

### 2.1. HPS Patients

For this study 31 HPS patients between the ages of 18 and 59 years were referred by their physicians to the University of New Mexico Health Science Center between 1995 and 2001 for treatment of suspected hantavirus infection. Written informed consent was obtained according to guidelines on human experimentation of the U.S. DHHS, and the study was approved by the Human Research Review Committee of the University of New Mexico. Clinical diagnosis was provisionally made by peripheral smear criteria [1] and confirmed by *N*-protein immunoblot assay [41]. As previously described [42], severe disease (*N* = 20) was defined as a requirement for mechanical ventilation (ratio of partial pressure of arterial oxygen/fraction of inspired oxygen <100). Moderately severe disease (*N* = 2) was defined as the need for inspired oxygen via continuous positive airway pressure (CPAP). Mild disease (*N* = 9) was defined by the need for oxygen delivered by nasal prongs only or no need for oxygen administration. Eight subjects with severe disease developed noncardiogenic shock unrelieved by intravenous vasopressor therapy and were treated with extracorporeal membrane oxygenation (ECMO) [43]. 

### 2.2. HPS Patient Samples

Venous blood was collected in EDTA tubes, centrifuged to separate plasma within one hour of collection, and stored at −80°C until analysis. Peripheral blood mononuclear cells (PBMC) were separated from arterial blood on Ficoll-Hypaque cushions, washed twice in PBS, and cryopreserved in 20% fetal calf serum, 10% DMSO at −70°C. Pulmonary edema fluid (PEF) was collected aseptically by deep aspiration from endotracheal tubes, centrifuged to remove cellular debris, and stored at −80°C. Pulmonary edema fluid (PEF) samples (*N* = 25) were collected from three patients, EDTA plasma samples (*N* = 56) were received from 31 cases, peripheral blood mononuclear cell samples (PBMCs) (*N* = 30) were collected from the blood of 21 patients and 7 serum samples were received from 4 patients. PBMCs were archived at 1.0–2.0 × 10^7^ cells/mL at −80°C and lysed completely after a secondary frosting-defrosting procedure. The lysates did not contain whole PBMC cells and the cells could not be counted prior to assay [1]. VEGF levels are reported as pg/10^7^ cells. Control samples used were derived from 13 plasma and sera samples collected from healthy individuals. Plasma VEGF levels were used as internal controls for PEF samples and control PBMC samples were collected from 6 healthy persons. All samples were coded and assayed without knowledge of clinical outcome. 

### 2.3. ELISA Assay

VEGF levels in samples were blindly assessed using a quantitative sandwich ELISA performed with the Human VEGF ELISA kit (Thermo Scientific) with a sensitivity of VEGF detection >8 pg/mL. Briefly, VEGF levels were determined following capture with a solid phase monoclonal antibody to VEGF_165_ IgG and detected using a biotin-conjugated polyclonal rabbit antibody against recombinant human VEGF_165_. The assay was performed according to the manufacture's protocol and concentrations are reported as pg/mL. Data are presented as the results of two independent blinded ELISAs. 

### 2.4. Statistical Analysis

Results were derived from two independent blinded experiments and presented as the mean ±SEM with *P* < 0.05 considered significant. Multiple group comparisons were made by one-way ANOVA. Two-way comparisons were performed by two-tailed, impaired Student's *t*-test. All analysis were performed using GraphPad Prism software version 5.0.3.

## 3. Results

### 3.1. VEGF in Pulmonary Edema Fluid

**Figure 1:**
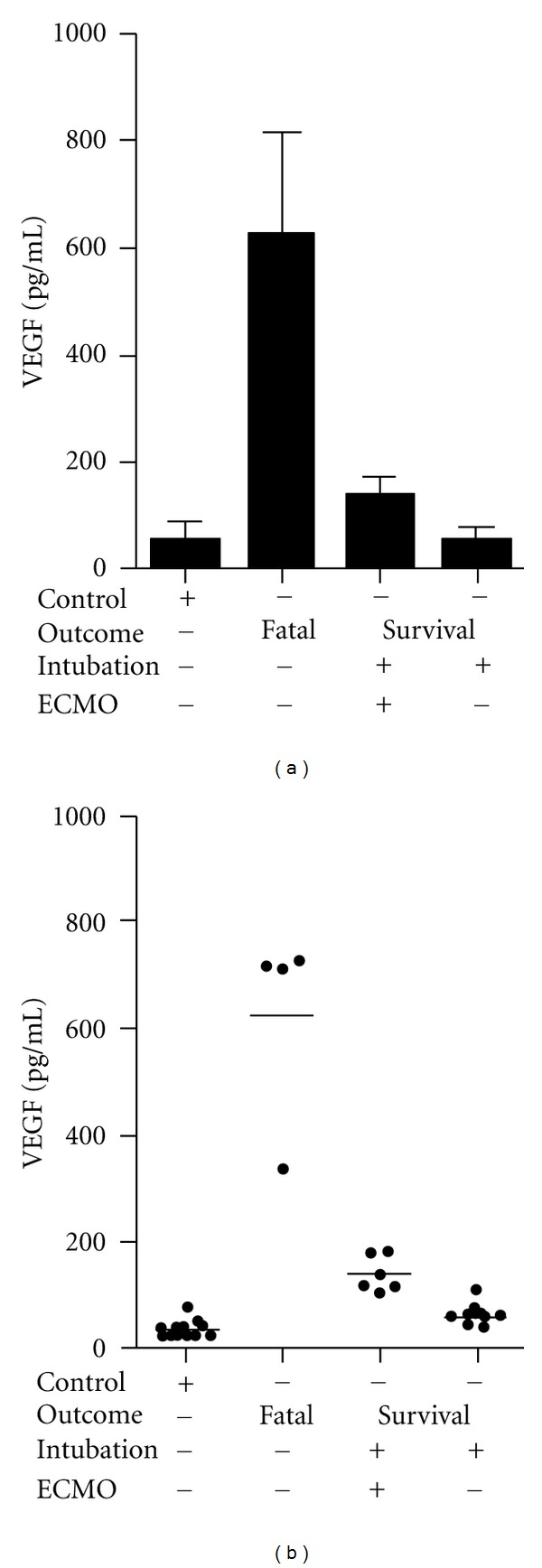
VEGF Levels in HPS Patient Pulmonary Edema Fluid. (a) Coded PEF samples from three patients were taken on the first and second days of hospitalization and tested by ELISA. Case 1 samples (*N* = 4) were taken at ~1 hour intervals from a patient presenting with severe HPS who died 6 hours after hospitalization. Case 2 samples (*N* = 6) were from a patient diagnosed with severe HPS who was intubated, ECMO-treated, and survived. Case 3 samples (*N* = 14) were from a patient with moderate HPS who was intubated and survived. Control VEGF levels are derived from 13 plasma samples from healthy individuals. Results from two independent ELISAs are presented and expressed as the mean SD. Differences between VEGF levels from the severe fatal patient were significantly different from those of severe ECMO-treated, moderate HPS or controls (*P* = 0.001) as determined by 1 way ANOVA test. VEGF levels of severe ECMO-treated patients were not significantly different from moderate HPS patients (*P* > 0.05), however VEGF levels from severe ECMO-treated patients were significantly different from control (*P* = 0.01). (b) Individual PEF VEGF levels from patients are presented.

HPS patient pulmonary edema fluid (PEF) samples are rare since they were not routinely stored. However, twenty three serial samples of pulmonary edema fluid (PEF) were available from three HPS patients which we retrospectively analyzed for VEGF levels. *HPS Patient 1*: four PEF samples were taken on the first day of hospitalization at ~1 hour intervals from a patient with severe HPS, who died hours later. *HPS Patient 2*: six PEF samples were taken on the first day of hospitalization from a second patient with severe HPS, who survived after intubation and extracorporeal membrane oxygenation (ECMO). *HPS Patient 3*: thirteen PEF samples were taken on the first and second day after hospitalization from a third patient with moderate-mild HPS who survived after a brief intubation. We found the highest PEF VEGF levels (mean 625 ± 95 pg/mL) in the fatal HPS case (Figure 1, Table 1), and VEGF levels in patient 1 were approximately 4-fold higher than in the ECMO-treated HPS patient (140.8 pg/mL). Patient 1 VEGF levels were 10-fold higher than the VEGF levels of the HPS patient with moderate-mild disease (mean 60.0 pg/mL) or comparable patient control samples (52.4 pg/mL). The number of PEF samples collected from three patients is a sample limitation that does not allow extrapolating data to all HPS cases. However, PEF samples from three patients with different HPS manifestations suggest that elevated VEGF levels correlate with HPS severity.

### 3.2. Enhanced VEGF Levels in PBMCs

**Figure 2:**
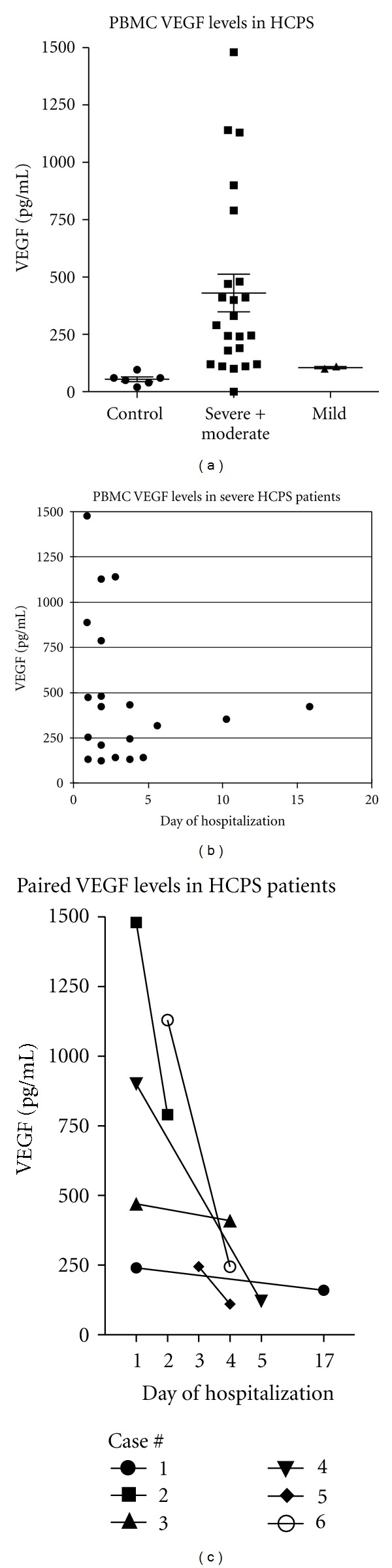
PBMC VEGF Levels from HPS Patients. VEGF present in PBMC lysates was measured as in Figure 1. (a) Individual VEGF levels in PBMCs are presented. Samples from severe cases (*N* = 24), moderate (*N* = 2), mild (*N* = 2), and control (*N* = 6) were analyzed by 1 way ANOVA. VEGF levels in severe and moderate severe cases were not significantly different (*P* > 0.05) (see Table 1) and are presented as one group. PBMC VEGF levels were significantly different between severe/moderate and mild or control groups (*P* < 0.001). (b) PBMC VEGF levels from patients with severe (20) HPS are presented (*P* < 0.001) relative to their time of collection (samples collected 1–3 days or 4–17 days after admission). A two-tailed *t*-test shows significantly (*P* = 0.03) higher VEGF level at early days after hospitalization. (c) Paired patient PBMC samples from 6 patients with severe HPS were taken during the course of disease and analyzed for VEGF levels. VEGF levels from samples collected 1–3 days compared to 4–17 days after admission were significantly higher at early stages of disease (*P* = 0.01 using two-tailed *t*-test).

We further studied PBMC-derived VEGF levels present in twenty-four HPS patient samples including: 24 with severe HPS, 2 moderate-severe cases, and 2 mild HPS patients. Mean VEGF levels in PBMCs from severe, moderate-severe, and mild HPS patient samples were 465.±85.7, 325 ± 075.0, and 105 ± 05.0 pg/mL, respectively. Differences in VEGF levels from severe and moderate cases versus control are statistically significant, *P* < 0.001 and *P* < 0.01, respectively, while VEGF levels in mild cases did not differ significantly from controls (*P* > 0.05) (Figure 2(a), Table 1). PBMC VEGF levels were highest during the first 3 days of hospitalization (*n* = 16, mean 526 ± 108.5) compared to samples taken 4–17 days after admission (*n* = 11, mean 217 ± 39.6; *P* = 0.03) and were found to decrease over time (Figure 2(b)). This observation was confirmed by VEGF levels in paired samples of 6 HPS patients taken in the same intervals after hospitalization: 1–3 days (*n* = 6, mean 797.5 ± 200.2) versus 4–17 days (*n* = 6, mean 212.3 ± 36.9; *P* = 0.01) (Figure 2(c)). 

### 3.3. VEGF Levels in Plasma and Sera

**Figure 3:**
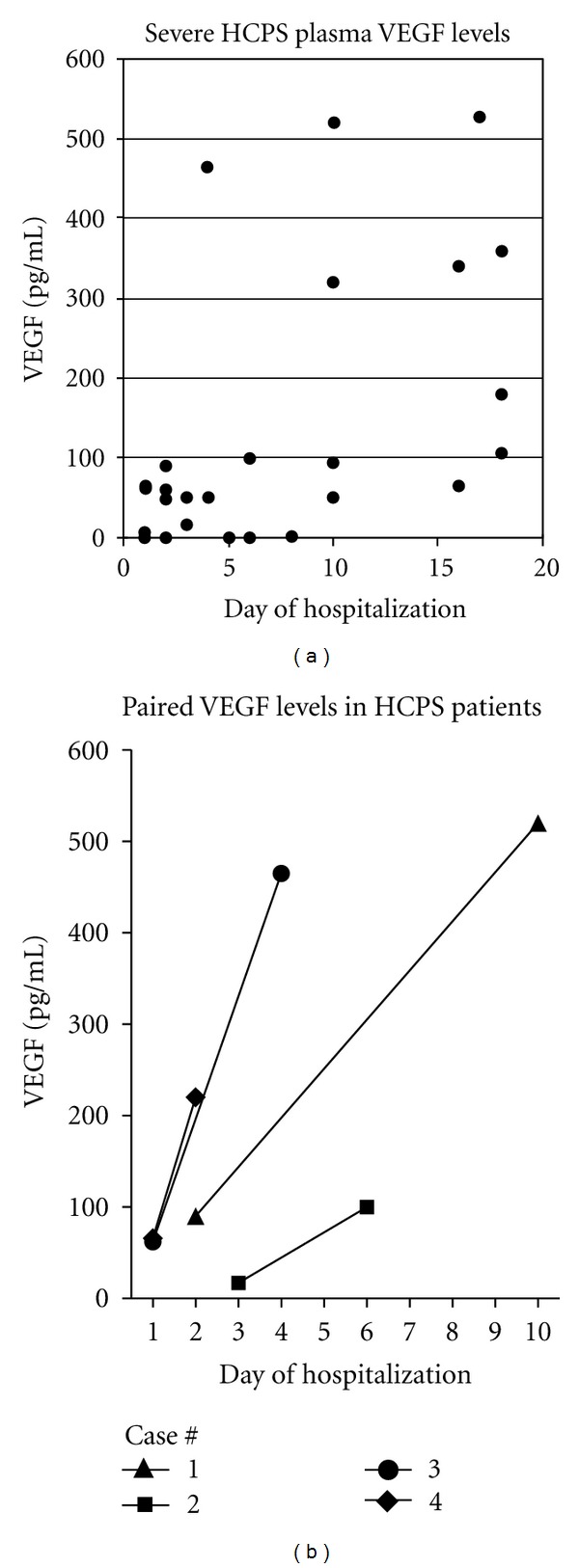
Plasma VEGF Levels of HPS Patients. (a) Plasma VEGF levels were analyzed from patients with severe (*N* = 30) forms of HPS as in Figure 1. Group statistical analysis was made by comparing groups relative to their time of collection (1–5 days compared to 6–10, or 11–20 days) using one-way ANOVA. VEGF levels from samples taken 1–5 days after-admission (*N* 12; mean 86 ± 34.8 mg/mL) were significantly (*P* < 0.05) lower than samples taken 11–20 days (*N* = 6; mean 262.2 ± 67.5 pg/mL) after-admission but not significant compared to samples taken 6–10 days or controls. Differences in plasma VEGF levels between severe and mild HPS patients were statistically insignificant (*P* > 0.05). (b) Paired VEGF levels from 4 HPS patient plasma samples are presented.

Serum VEGF levels were not statistically different between controls, mild, or severe HPS patient samples (3 severe and 4 mild cases) (*P* > 0.05) (Table 1). In contrast to PEF and PBMC samples, we found low-plasma VEGF levels in severe HPS cases during the first 3 days of hospitalization (*n* = 15, mean 30.4 ± 8.1), and an increase in plasma VEGF levels during recovery (*n* = 17, mean 212 ± 49.7; *P* = 0.001) (Figures 3(a), and 3(b); Table 1). Plasma VEGF levels were, respectively, 2 to >3 fold higher in mild and severe HPS patients than in controls. These results are consistent with localized rather than systemic permeability responses of VEGF and findings that released VEGF reportedly binds receptors within 0.5 mm of its release and is inactivated by circulating soluble receptors [36, 44]. 

### 3.4. VEGF Levels in Parallel Samples

We blindly tested parallel samples, but after decoding noticed that a few samples that were taken from the same patients. In two cases, elevated VEGF levels were correspondingly observed in PEF and PBMC samples: severe HPS: 350 and 245 pg/mL, respectively moderate severe case 180 and 90 pg/mL; respectively. Additional positively correlated VEGF findings were detected in parallel PBMC and plasma samples from 7 patients (mean 229.2 ± 47.1 pg/mL and 79.3 ± 36.9 pg/mL; *P* = 0.03).

## 4. Discussion

Pulmonary symptoms of HPS patients are characterized by hypoxia and extensive capillary leakage resulting in acute bilateral pulmonary edema [2, 5–7, 22, 42, 45]. Hypoxia induces pulmonary epithelial and endothelial cells to secrete VEGF [32, 34–37, 39, 40, 46, 47], and VEGF acts on the endothelium to stimulate growth and dissociate adherence junctions between endothelial cells [32, 36, 46, 48–51]. This permits vascular remodeling, vessel repair, and angiogenesis, but can also locally increase capillary permeability [32, 36, 39, 44, 46, 48, 49, 52]. A VEGF-HIF-1*α* amplification loop is responsible for high-altitude-induced pulmonary edema, and over expressing VEGF in the lung causes pulmonary edema [33–35, 37–40, 52, 53]. Conversely, genetic delivery of antiVEGF antibody or antagonizing VEGF responses suppresses pulmonary edema in experimental animals [37, 54–57]. 

ECMO reduces the progression of respiratory failure and the mortality of HPS from ~75% to 35–40% [2, 22, 58] suggesting a role for hypoxia-induced VEGF in HPS edema [34, 35, 39, 40, 47, 52, 59]. Pulmonary VEGF is associated with localized pathogenesis and as a cause of high-altitude pulmonary edema in response to reduced oxygen levels [6, 33, 33, 35–37, 39, 40, 47, 52]. Activated pulmonary PBMCs are increased in HPS patients and PBMCs also secrete VEGF in response to hypoxia suggesting a potential mechanism by which localized VEGF immune responses to hantavirus could contribute to disease [5–7, 60, 61]. Our findings indicate the presence of elevated VEGF in PEFs from severe and moderate HPS cases that are 4–10 fold above VEGF levels in patients with mild HPS and similar to PEF VEGF levels from patients with hydrostatic edema (median 799 pg/mL) or acute lung injury (median 501 pg/mL) [39, 40, 52, 62]. Our findings also indicate that HPS patient PBMCs contain high VEGF levels at acute stages 1–5 days after hospitalization, which diminished over time in paired patient samples (Figures 2(b), and 2(c)). Although samples available were insufficient to analyze viremia here, these early VEGF responses occur with similar timing to the high-level viremia previously reported within HPS patients [63]. Collectively, these findings suggest that the reduced mortality observed following HPS patient oxygenation may be at least partly derived from inhibiting VEGF/HIF-1*α* responses which result in a concomitant reduction in hypoxia-directed pulmonary edema [34, 35, 37, 40, 47].

VEGF acts within millimeters of its release to prevent systemic capillary permeability [44], and serum VEGF is inactivated by binding to circulating soluble receptors [32, 36, 39, 52]. While localized PEF and PBMCs had high VEGF levels during acute HPS stages we found that circulating plasma and serum VEGF levels were low in severe HPS patients during acute HPS stages (1–5 days after hospitalization). In contrast, circulating serum and plasma VEGF levels only increased 11–20 days after-admission (Figures 3(a), and 3(b)). Increased circulating VEGF at late times after infection is consistent with vascular remodeling and repair that occurs during recovery phases of other causes of acute pulmonary edema and may coincide with HPS convalescence [32, 33, 36, 39, 44, 46, 48, 49, 52, 64]. These findings are similar to studies of patients with high-altitude-induced pulmonary edema where pulmonary VEGF levels are associated with acute disease and plasma levels of VEGF only become elevated during recovery [33]. These findings suggest the direct involvement of localized PEF and PBMC VEGF responses in acute HPS pathogenesis and the potential for circulating VEGF to be a sign of patient recovery. 

Hantaviruses infect endothelial cells in pulmonary capillary beds [2] and cause hypoxia in HPS patients [2, 5–7, 45]. Finding increased VEGF in PEFs and PBMCs from HPS cases suggests hypoxia-induced pulmonary VEGF induction as a potential edemagenic mechanism [33, 35, 39, 40, 46, 52]. This data is supported by *in vitro* results demonstrating that hantavirus-infected endothelial cells are hyperresponsive to the permeabilizing effects of VEGF and that blocking VEGFR2-Src signaling responses inhibits permeability [17, 18, 24, 30]. This mechanism is further linked to the ability of pathogenic hantaviruses to block *α*_*v*_ *β*_3_ integrin functions, which normally restrict VEGFR2 permeabilizing responses [18–21, 27, 28]. Thus HPS patient hypoxia in combination with hantavirus-infected VEGF-hyperresponsive endothelial cells is likely to contribute to acute pulmonary edema. Moreover, these results suggest that pathway specific VEGF inhibitors may be clinically relevant and used in tandem with ECMO to reduce the severity of HPS. 

## Figures and Tables

**Table 1:** VEGF level in HCPS patients samples.

Sample	*n*	VEGF positive	VEGF pg/mL		*P* versus control
			Range	Mean
PEF					
Severe lethal	4	4	340–730	625.0 ± 95.1	<0.001
Severe survival	6	6	105–180	140.8 ± 38.0	<0.01
Moderate severe	14	14	40–110	60.0 ± 5.1	>0.05
Control	13	13	20–80	52.4 ± 6.1	
PBMC					
Severe	24	23	<8–1,480	465.0 ± 85.7	<0.001
Moderate	2	2	250–400	325.0 ± 75.0	<0.01
Mild	2	2	100–110	105.0 ± 5.0	>0.05
Control	6	6	10–150	61.5 ± 7.4	
Plasma					
Severe	39	30	10–527	174.4 ± 31.6	<0.01
Mild	17	8	10–310	94.2 ± 23.4	>0.05
Control	13	13	20–80	52.4 ± 6.1	
Serum					
Severe	3	3	50–60	51.3 ± 3.7	>0.05
Mild	4	2	<8–110	32.5 ± 26.2	>0.05
Control	13	13	22–110	47.2 ± 17.5	

## References

[1] Koster F, Foucar K, Hjelle B (2001). Rapid presumptive diagnosis of hantavirus cardiopulmonary syndrome by peripheral blood smear review. *American Journal of Clinical Pathology*.

[2] Koster F, Mackow ER (2012). Pathogenesis of the hantavirus pulmonary syndrome. *Future Virology*.

[3] Nichol ST, Spiropoulou CF, Morzunov S (1993). Genetic identification of a hantavirus associated with an outbreak of acute respiratory illness. *Science*.

[4] Schmaljohn C, Hjelle B (1997). Hantaviruses: a global disease problem. *Emerging Infectious Diseases*.

[5] Zaki SR, Greer PW, Coffield LM (1995). Hantavirus pulmonary syndrome: pathogenesis of an emerging infectious disease. *American Journal of Pathology*.

[6] Duchin JS, Koster FT, Peters CJ (1994). Hantavirus pulmonary syndrome: a clinical description of 17 patients with a newly recognized disease. *New England Journal of Medicine*.

[7] Nolte KB, Feddersen RM, Foucar K (1995). Hantavirus pulmonary syndrome in the United States: a pathological description of a disease caused by a new agent. *Human Pathology*.

[8] Kanerva M, Mustonen J, Vaheri A (1998). Pathogenesis of puumala and other hantavirus infections. *Reviews in Medical Virology*.

[9] Kilpatrick ED, Terajima M, Koster FT, Catalina MD, Cruz J, Ennis FA (2004). Role of specific CD8+ T cells in the severity of a fulminant zoonotic viral hemorrhagic fever, hantavirus pulmonary syndrome. *Journal of Immunology*.

[10] Krakauer T, Leduc JW, Krakauer H (1995). Serum levels of tumor necrosis factor-*α*, interleukin-1, and interleukin-6 in hemorrhagic fever with renal syndrome. *Viral Immunology*.

[11] Mackow ER, Gavrilovskaya IN (2009). Hantavirus regulation of endothelial cell functions. *Thrombosis and Haemostasis*.

[12] Maes P, Clement J, Groeneveld PHP, Colson P, Huizinga TWJ, Van Ranst M (2006). Tumor necrosis factor-*α* genetic predisposing factors can influence clinical severity in nephropathia epidemica. *Viral Immunology*.

[13] Mori M, Rothman AL, Kurane I (1999). High levels of cytokine-producing cells in the lung tissues of patients with fatal hantavirus pulmonary syndrome. *Journal of Infectious Diseases*.

[14] Sundstrom JB, McMullan LK, Spiropoulou CF (2001). Hantavirus infection induces the expression of RANTES and IP-10 without causing increased permeability in human lung microvascular endothelial cells. *Journal of Virology*.

[15] Temonen M, Mustonen J, Helin H, Pasternack A, Vaheri A, Holthöfer H (1996). Cytokines, adhesion molecules, and cellular infiltration in nephropathia epidemica kidneys: an immunohistochemical study. *Clinical Immunology and Immunopathology*.

[16] Khaiboullina SF, Netski DM, Krumpe P, Jeor SCS (2000). Effects of tumor necrosis factor alpha on Sin Nombre virus infection in vitro. *Journal of Virology*.

[17] Gavrilovskaya IN, Gorbunova EE, Mackow ER (2010). Pathogenic hantaviruses direct the adherence of quiescent platelets to infected endothelial cells. *Journal of Virology*.

[18] Gavrilovskaya IN, Gorbunova EE, Mackow NA, Mackow ER (2008). Hantaviruses direct endothelial cell permeability by sensitizing cells to the vascular permeability factor VEGF, while angiopoietin 1 and sphingosine 1-phosphate inhibit hantavirus-directed permeability. *Journal of Virology*.

[19] Gavrilovskaya IN, Peresleni T, Geimonen E, Mackow ER (2002). Pathogenic hantaviruses selectively inhibit *β*3 integrin directed endothelial cell migration. *Archives of Virology*.

[20] Raymond T, Gorbunova E, Gavrilovskaya IN, Mackow ER (2005). Pathogenic hantaviruses bind plexin-semaphorin-integrin domains present at the apex of inactive, bent *α*v*β*3 integrin conformers. *Proceedings of the National Academy of Sciences of the United States of America*.

[21] Robinson SD, Reynolds LE, Wyder L, Hicklin DJ, Hodivala-Dilke KM (2004). *β*3-integrin regulates vascular endothelial growth factor-A-dependent permeability. *Arteriosclerosis, Thrombosis, and Vascular Biology*.

[22] Chang B, Crowley M, Campen M, Koster F (2007). Hantavirus cardiopulmonary syndrome. *Seminars in Respiratory and Critical Care Medicine*.

[23] Geimonen E, Neff S, Raymond T, Kocer SS, Gavrilovskaya IN, Mackow ER (2002). Pathogenic and nonpathogenic hantaviruses differentially regulate endothelial cell responses. *Proceedings of the National Academy of Sciences of the United States of America*.

[24] Gorbunova E, Gavrilovskaya IN, Mackow ER (2010). Pathogenic hantaviruses Andes virus and Hantaan virus induce adherens junction disassembly by directing vascular endothelial cadherin internalization in human endothelial cell. *Journal of Virology*.

[25] Khaiboullina SF, Rizvanov AA, Otteson E, Miyazato A, Maciejewski J, Jeor SS (2004). Regulation of cellular gene expression in endothelial cells by Sin Nombre and Prospect Hill viruses. *Viral Immunology*.

[26] Yanagihara R, Silverman DJ (1990). Experimental infection of human vascular endothelial cells by pathogenic and nonpathogenic hantaviruses. *Archives of Virology*.

[27] Hodivala-Dilke KM, McHugh KP, Tsakiris DA (1999). *β*3-integrin-deficient mice are a model for Glanzmann thrombasthenia showing placental defects and reduced survival. *Journal of Clinical Investigation*.

[28] Reynolds AR, Reynolds LE, Nagel TE (2004). Elevated Flk1 (vascular endothelial growth factor receptor 2) signaling mediates enhanced angiogenesis in *β*3-integrin-deficient mice. *Cancer Research*.

[29] Gavrilovskaya IN, Shepley M, Shaw R, Ginsberg MH, Mackow ER (1998). *β*3 integrins mediate the cellular entry of hantaviruses that cause respiratory failure. *Proceedings of the National Academy of Sciences of the United States of America*.

[30] Gorbunova EE, Gavrilovskaya IN, Pepini T, Mackow ER (2011). VEGFR2 and Src kinase inhibitors suppress Andes virus-induced endothelial cell permeability. *Journal of Virology*.

[31] Pepini T, Gorbunova EE, Gavrilovskaya IN, Mackow JE, Mackow ER (2010). Andes virus regulation of cellular microRNAs contributes to hantavirus-induced endothelial cell permeability. *Journal of Virology*.

[32] Dvorak HF, Brown LF, Detmar M, Dvorak AM (1995). Vascular permeability factor/vascular endothelial growth factor, microvascular hyperpermeability, and angiogenesis. *American Journal of Pathology*.

[33] Hanaoka M, Droma Y, Naramoto A, Honda T, Kobayashi T, Kubo K (2003). Vascular endothelial growth factor in patients with high-altitude pulmonary edema. *Journal of Applied Physiology*.

[34] Berger MM, Hesse C, Dehnert C (2005). Hypoxia impairs systemic endothelial function in individuals prone to high-altitude pulmonary edema. *American Journal of Respiratory and Critical Care Medicine*.

[35] Dehler M, Zessin E, Bärtsch P, Mairbäurl H (2006). Hypoxia causes permeability oedema in the constant-pressure perfused rat lung. *European Respiratory Journal*.

[36] Dvorak HF (2010). Vascular permeability to plasma, plasma proteins, and cells: an update. *Current Opinion in Hematology*.

[37] Stenmark KR, Fagan KA, Frid MG (2006). Hypoxia-induced pulmonary vascular remodeling: cellular and molecular mechanisms. *Circulation Research*.

[38] Tang N, Wang L, Esko J (2004). Loss of HIF-1*α* in endothelial cells disrupts a hypoxia-driven VEGF autocrine loop necessary for tumorigenesis. *Cancer Cell*.

[39] Kaner RJ, Crystal RG (2004). Pathogenesis of high altitude pulmonary edema: does alveolar epithelial lining fluid vascular endothelial growth factor exacerbate capillary leak?. *High Altitude Medicine and Biology*.

[40] Kaner RJ, Ladetto JV, Singh R, Fukuda N, Matthay MA, Crystal RG (2000). Lung overexpression of the vascular endothelial growth factor gene induces pulmonary edema. *American Journal of Respiratory Cell and Molecular Biology*.

[41] Hjelle B, Jenison S, Torrez-Martinez N (1997). Rapid and specific detection of Sin Nombre virus antibodies in patients with hantavirus pulmonary syndrome by a strip immunoblot assay suitable for field diagnosis. *Journal of Clinical Microbiology*.

[42] Hallin GW, Simpson SQ, Crowell RE (1996). Cardiopulmonary manifestations of hantavirus pulmonary syndrome. *Critical Care Medicine*.

[43] Crowley MR, Katz RW, Kessler R (1998). Successful treatment of adults with severe Hantavirus pulmonary syndrome with extracorporeal membrane oxygenation. *Critical Care Medicine*.

[44] Dvorak HF, Sioussat TM, Brown LF (1991). Distribution of vascular permeability factor (vascular endothelial growth factor) in tumors: concentration in tumor blood vessels. *Journal of Experimental Medicine*.

[45] Bustamante EA, Levy H, Simpson SQ (1997). Pleural fluid characteristics in hantavirus pulmonary syndrome. *Chest*.

[46] Dvorak HF (2006). Discovery of vascular permeability factor (VPF). *Experimental Cell Research*.

[47] Pham I, Uchida T, Planes C (2002). Hypoxia upregulates VEGF expression in alveolar epithelial cells in vitro and in vivo. *American Journal of Physiology-Lung Cellular and Molecular Physiology*.

[48] Dejana E, Orsenigo F, Lampugnani MG (2008). The role of adherens junctions and VE-cadherin in the control of vascular permeability. *Journal of Cell Science*.

[49] Gavard J, Gutkind JS (2006). VEGF Controls endothelial-cell permeability promoting *β*-arrestin-dependent Endocytosis VE-cadherin. *Nature Cell Biology*.

[50] Lampugnani MG, Dejana E (2007). The control of endothelial cell functions by adherens junctions. *Novartis Foundation Symposium*.

[51] Lampugnani MG, Dejana E (2007). Adherens junctions in endothelial cells regulate vessel maintenance and angiogenesis. *Thrombosis Research*.

[52] Kaner RJ, Crystal RG (2001). Compartmentalization of vascular endothelial growth factor to the epithelial surface of the human lung. *Molecular Medicine*.

[53] Hopkins SR, Garg J, Bolar DS, Balouch J, Levin DL (2005). Pulmonary blood flow heterogeneity during hypoxia and high-altitude pulmonary edema. *American Journal of Respiratory and Critical Care Medicine*.

[54] Thurston G, Rudge JS, Ioffe E (2000). Angiopoietin-1 protects the adult vasculature against plasma leakage. *Nature Medicine*.

[55] Thurston G, Suri C, Smith K (1999). Leakage-resistant blood vessels in mice transgenically overexpressing angiopoietin-1. *Science*.

[56] Wang Y, Pampou S, Fujikawa K, Varticovski L (2004). Opposing effect of angiopoietin-1 on VEGF-mediated disruption of endothelial cell-cell interactions requires activation of PKC*β*. *Journal of Cellular Physiology*.

[57] Watanabe M, Boyer JL, Crystal RG (2009). Genetic delivery of bevacizumab to suppress vascular endothelial growth factor-induced high-permeability pulmonary edema. *Human Gene Therapy*.

[58] Dietl CA, Wernly JA, Pett SB (2008). Extracorporeal membrane oxygenation support improves survival of patients with severe Hantavirus cardiopulmonary syndrome. *Journal of Thoracic and Cardiovascular Surgery*.

[59] Mukhopadhyay D, Tsiokas L, Zhou XM, Foster D, Brugge JS, Sukhatme VP (1995). Hypoxic induction of human vascular endothelial growth factor expression through c-Src activation. *Nature*.

[60] Salven P, Orpana A, Joensuu H (1999). Leukocytes and platelets of patients with cancer contain high levels of vascular endothelial growth factor. *Clinical Cancer Research*.

[61] Zhang J, Silva T, Yarovinsky T (2010). VEGF blockade inhibits lymphocyte recruitment and ameliorates immune-mediated vascular remodeling. *Circulation Research*.

[62] Ware LB, Kaner RJ, Crystal RG (2005). VEGF levels in the alveolar compartment do not distinguish between ARDS and hydrostatic pulmonary oedama. *European Respiratory Journal*.

[63] Xiao R, Yang S, Koster F, Ye C, Stidley C, Hjelle B (2006). Sin Nombre viral RNA load in patients with hantavirus cardiopulmonary syndrome. *Journal of Infectious Diseases*.

[64] Gracia F, Armien B, Simpson SQ (2010). Convalescent pulmonary dysfunction following hantavirus pulmonary syndrome in panama and the United States. *Lung*.

